# Feeding habits of mosquitoes (Diptera: Culicidae) in an area of sylvatic transmission of yellow fever in the state of São Paulo, Brazil

**DOI:** 10.1186/s40409-015-0005-z

**Published:** 2015-03-20

**Authors:** Luis Filipe Mucci, Rubens Pinto Cardoso Júnior, Marcia Bicudo de Paula, Sirle Abdo Salloum Scandar, Márcio Lunardeli Pacchioni, Aristides Fernandes, Cleide Aschenbrenner Consales

**Affiliations:** Laboratory of Biology and Ecology of Mosquitoes, Superintendence for Endemic Disease Control, State Health Secretariat, Taubaté, São Paulo State Brazil; Regional Service 08, Superintendence for Endemic Disease Control, State Health Secretariat, São José do Rio Preto, São Paulo State Brazil; Department of Epidemiology, School of Public Health, University of São Paulo (USP), São Paulo, São Paulo State Brazil; Regional Service 09, Superintendence for Endemic Disease Control, State Health Secretariat, Araçatuba, São Paulo State Brazil; Diagnostics Section, Pasteur Institute, State Health Secretariat, São Paulo, São Paulo State Brazil

**Keywords:** Feeding habit, Sylvatic yellow fever, Neotropical mosquitoes, Non-human primate, Forest fragmentation

## Abstract

**Background:**

The reintroduction of sylvatic yellow fever in the state of São Paulo after about six decades was confirmed in the Northwestern region in 2000, where in 2008 there also occurred an important epizootic. The purpose of this study was to investigate the feeding habits of culicids potentially involved in the sylvatic transmission of the virus in this region.

**Methods:**

Specimens were collected in 24 forested localities at ground level with hand nets and mouth aspirators. Collections were made quarterly between October 2006 and July 2008 during daylight hours. Blood-meal identification was carried out in mosquitoes of the tribes Aedini, Mansoniini and Sabethini. The biotin/avidin sandwich ELISA was employed to determine six source types: bird, bovine, equine, rat, human and monkey.

**Results:**

A total of 24,879 females of the three tribes were obtained, 245 (0.98%) of which were engorged. The presence of three different blood sources per engorged female was the predominant situation, and included 35.10% of the total of samples processed. Samples with two or four different sources were represented by 25.31% and 25.71%, of the specimens, respectively, while just 9.39% had only one type and 1.22%, five different sources. *Aedes scapularis*, *Ae. serratus* (Group), *Psorophora albigenu* and *Ps. ferox* were the most abundant species and accounted for about 95% of the engorged specimens. Of the principal vector species, *Haemagogus janthinomys/capricornii* was found with bird, bovine and primate blood. These sources were predominant and alternated top ranking as the most frequent source according to the mosquito species and collection site. In general, primate blood was the most prevalent source.

**Conclusions:**

The human population of the region visits this ecotone frequently, which indicates the need for the periodical assessment of vaccination coverage against yellow fever. The frequency of non-human primate blood source in mosquito species that show minor vector importance in yellow fever virus transmission deserves attention. The eclectic feeding habits and some aspects of the interactions between potential vectors and reservoirs of yellow fever may be associated with the habitat fragmentation characteristic of the region. We recommend that further studies on the capacity and vector competence be performed on secondary vectors in extra-Amazonian region.

**Electronic supplementary material:**

The online version of this article (doi:10.1186/s40409-015-0005-z) contains supplementary material, which is available to authorized users.

## Background

The last 20 years have been characterized by the expansion of sylvatic transmission of yellow fever beyond the Amazonian biome in Brazil [1]. This situation represents a serious challenge for public health services, especially in relation to the improvement of surveillance systems, the defining of strategies for the immunization of the susceptible population and the adoption of preventive measures to avoid urban transmission [2-6].

Within this scenario, the hypotheses relating to the introduction and circulation of the virus have been called into question in view of the observation of the distinct aspects of the localities affected in terms of vector fauna, isolation of viruses and the epidemiological or epizootic characteristics of the outbreaks. Generally speaking, non-human primates have always been considered the main reservoirs of the virus in the natural environment [1,7]. However, the participation of other mammals in the maintenance of the virus and the influence of the double vector-reservoir role of the mosquitoes in the disease cycle are discussed [1,8,9]. Furthermore, it was recently suggested a potential mediation of human migrations in virus dispersion based on genetic divergence in Brazilian strains [10].

As to the vector importance of the Culicidae, *Haemagogus janthinomys* and *Hg. leucocelaenus* are actually considered the main vectors of the yellow-fever virus in the country [1]. Other species such as *Hg. capricornii*, *Hg. albomaculatus*, *Hg. spegazzinii*, *Hg. tropicalis*, *Sabethes chloropterus*, *Sa. glaucodaemon*, *Sa. soperi*, *Sa. cyaneus, Sa. quasicyaneus, Ae. serratus, Aedes fulvus*, *Ae. scapularis, Psorophora albipes* and *Ps. ferox* have generally been considered locally important or auxiliary vectors, and for some of these species, viral isolation was recorded only once [11-14].

Studying the Culicidae feeding habits has been proven fundamental in the elucidation of the yellow-fever epidemiological chain [15-17]. The role of some of these vectors as a link in the interchange of viruses between humans and other primates has long since been demonstrated, but it calls for a re-investigation in order to update our knowledge of regional “eco-epidemiologies” and expand research to cover other mosquito species and alternative feeding sources [18]. It is important to bear in mind that the dispersal and availability of vertebrate hosts, including humans and domestic animals, have been undergoing significant changes by virtue of the increase in agricultural and cattle-raising activities and the degradation of forest environments, which may be bringing adaptive pressure to bear on the subpopulations of these mosquitoes, raising the possibility of alterations related not only to the characteristics of their niches but also to their vector capacity [19].

In the present study we have sought to investigate the feeding habits of mosquitoes of the Aedini, Mansoniini and Sabethini tribes collected in forest environments adjacent to the main rivers of the northwestern region of São Paulo. The state where the reintroduction of yellow fever was confirmed in 2000 after an absence of about six decades, and the location in 2008 of one of the great epizootic outbreaks recently registered in Brazil [5,20].

**Figure 1:**
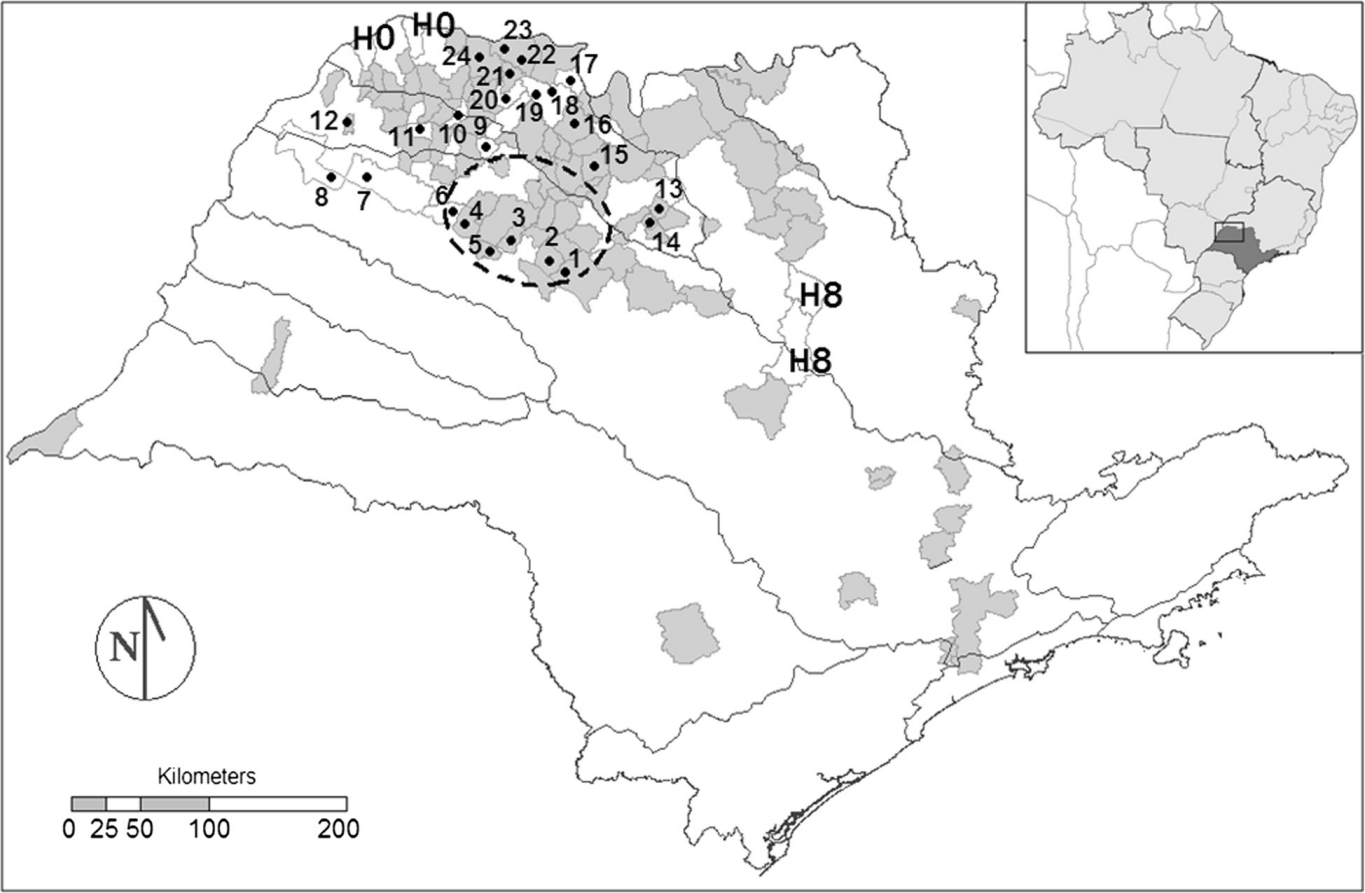
**Map of the state of São Paulo showing the collection sites and events related to yellow fever in 2000 and 2008.** Black dots represent the collection sites by number. The larger polygons represent the main river basins of the state. The smaller polygons not filled in indicate municipalities with entomological collections or autochthonous human cases of yellow fever in 2000 (H0) and 2008 (H8). When filled in gray, the area indicates deaths of monkeys in 2008. The dashed line represents the region where the epizootic outbreaks have been confirmed by Adolfo Lutz Institute/State Health Secretariat of São Paulo, Brazil.

Coincidently, these epizootics were simultaneous with the period of the field collection, when there was in the region a series of communications on the deaths of monkeys (n = 242) with clinical and epidemiological confirmation (n = 65) and laboratory confirmation (n = 4) for yellow fever [21], as well as the occurrence of two human cases in the municipalities of Luiz Antônio and São Carlos, toward the southeast (Figure 1).

## Methods

A total of 24 collection sites were selected in the northwestern region of the state of São Paulo, in 22 municipalities spread over the river basins of the Tietê, São José dos Dourados and Turvo (Figure 1). The area covers the territory of 167 municipalities with a total population of about 3.3 million inhabitants, according to the IBGE census of 2010. The entire study area is included in the “area with vaccine recommendation for yellow fever” determined by the Epidemiological Surveillance Center/Disease Control Coordination of the São Paulo State Health Secretariat. The estimated vaccine coverage for the period of 2003 to 2013 exhibits a predominance of subareas with rates between 60% and 90%, followed by those between 20% and 60%, and a few areas with rates over 90% [22].

The collections were undertaken within forests of secondary succession characteristics, represented by fragments of gallery forest, forested savannah (*Cerradão*), semi-deciduous seasonal forests and phytophysiognomy of contact between these types [23]. Variations were observed in the herbaceous and bush strata, in the canopy height (between 8 and 25 meters), and in the size of the forest fragments (from 6.50 to 2806.44 hectares) (Additional file 1). With regard to climate, except for the sites in the municipality of Riolândia which presented a humid subtropical climate (Cwa), the other sites were situated in the tropical savanna climate (Aw) in accordance with Köppen’s classification, both types having rainy summers and dry winters [23].

In order to cover the four seasons of the year, the collections were made quarterly in the months of October, January, April and July, between October 2006 and July 2007, at the sites located in the basins of the rivers Turvo and São José dos Dourados, and between October 2007 and July 2008 at those in the Tietê River basin. The captures were carried out on two consecutive days at each of the sites, in the period from 09:00 to 15:00 hours, with the participation of three collectors who walked through the interior of the forest making frequent stops to apprehend the specimens by approaching them with a hand-net catcher and mouth aspirator (Castro’s capturers). Individual protection equipment was used (a legionnaire’s cap, gloves and jacket) to avoid contact between the collectors and the mosquitoes, to ensure that the human blood found in the engorged females was not that of the collectors themselves. The specimens obtained were sacrificed in chloroform vapor and conditioned in entomological boxes each hour, when not engorged. The engorged females were placed individually in 1.5 mL cryotubes, preserved in the field in thermal boxes with ice and later conserved in a freezer at −20°C until they could be identified and processed.

Identification was undertaken individually to the taxonomic species level, following Consoli and Lourenço-de-Oliveira [24], Forattini [25] and Lane [26] whereas the name abbreviations used were those of Reinert [27]. A cold table was used for the identification of the frozen material; and the engorged females belonging to the Aedini, Mansoniini and Sabethini tribes were selected for investigation of the blood source. In this study we consider that *Aedes serratus* Group includes females of *Ae. serratus* and *Ae. serratus/aenigmaticus*.

The technique employed to determine the presence of ingested blood in the abdominal contents was the biotin/avidin sandwich enzyme-linked immunosorbent assay (ELISA) in accordance with the procedures used by Marassá *et al.* [17]. The six feeding sources tested were: bird, bovine, equine, human, monkey and rat. For counting the number of different blood sources, we considered five major groups by Family level, grouping humans and monkeys, to enable comparison with results of other studies that do not distinguish these two types.

In the remainder of the results, we considered the distinction between the two types of primates as follows: the human and monkey reagent samples were re-submitted to reaction with the IgG1 human subclass, reactivity to which confirmed the presence of human blood without, however, eliminating the presence of monkey blood, which was denominated primates (human + monkey). The samples reactive to human IgG (total) and subclass IgG1, but non-reactive to monkey were considered human (exclusively). Samples non-reactive to human IgG1 were considered to contain only monkey blood by the exclusion criterion, which indicates non-human primates (NHP).

## Results

A total of 25,395 mosquitoes of the Aedini, Mansoniini and Sabethini tribes were collected, 24,879 (97.97%) of which were female and 516 (2.03%) male, belonging to eight genera and 42 species, representing 45 taxa, including specimens identified to the subgenus level (Additional file 2). The females whose abdomens were distended with fresh blood, eggs, or mixed content numbered 358 (1.44%), 245 (0.98%) of which, with fresh blood (engorged), were selected and processed. Engorged females (samples) were obtained at 20 of the 24 sites investigated.

Of the principal vector species, only *Hg. janthinomys/capricornii* was found engorged, despite having been less abundant than *Hg. leucocelaenus* (n = 147 × n = 346 females). Of the 845 females of the genus *Sabethes*, belonging to the following species: *Sa. glaucodaemon* (n = 690), *Sa. albiprivus* (n = 100), *Sa. gymnothorax* (n = 27), *Sa. tridentatus* (n = 15), *Sa. chloropterus* (n = 9), *Sa. undosus/fabricii* (n = 3) and *Sabethes* spp*.* (n = 1) (Additional file 2), only two females of *Sa. glaucodaemon* presented a distended abdomen, though none was engorged.

*Aedes scapularis, Ae. serratus* (Group), *Ps. albigenu* and *Ps. ferox* were the most abundant species and accounted for about 95% of the engorged specimens. The rate of engorged females out of all females collected varied according to the collection sites for each species. For *Ae. scapularis* the rate varied from 0.20 to 11.54%, for *Ae. serratus* (Group) from 0.49 to 5.36% (site 3 with 100% of the two specimens captured), for *Ps. albigenu* from 0.25 to 14.29%, for *Ps. ferox* from 0.62 to 6.67% (Table 1) and for less abundant species from 0.14 to 2.27%, except for *Ae. fulvus*, which presented only one female collected and engorged (Additional file 3).

**Table 1:** **Number of collected and engorged females by collection site and municipality**

**Municipality**	**Collection site**	**Species**														
		***Ae. scapularis***			***Ae. serratus*** **(G)**			***Ps. albigenu***			***Ps. ferox***			**Others**		
		**Col.**	**Eng.**	**%E/C**	**Col.**	**Eng.**	**%E/C**	**Col.**	**Eng.**	**%E/C**	**Col.**	**Eng.**	**%E/C**	**Col.**	**Eng.**	**%E/C**
Novo Horizonte	1	1505	3	0.20	1	0	0.00	65	1	1.54	22	1	4.55	40	0	0.00
Sales	2	629	7	1.11	16	0	0.00	15	0	0.00	19	0	0.00	128	0	0.00
Ubarana	3	44	2	4.55	2	2	100	4	0	0.00	1	0	0.00	521	3	0.58
Zacarias	4	392	0	0.0	5	0	0.00	15	0	0.00	6	0	0.00	10	0	0.00
Barbosa	5	115	0	0.0	3	0	0.00	81	0	0.00	2	0	0.00	379	0	0.00
Buritama	6	226	3	1.33	3	0	0.00	12	0	0.00	5	0	0.00	5	0	0.00
Santo Antônio do Aracanguá	7	1125	10	0.89	0	0	0.00	1	0	0.00	1	0	0.00	11	0	0.00
Pereira Barreto	8	181	1	0.55	1	0	0.00	30	0	0.00	22	0	0.00	8	0	0.00
Sebastianópolis do Sul	9	828	19	2.29	59	0	0.00	705	24	3.40	58	0	0.00	12	0	0.00
Valentim Gentil	10	171	10	5.85	58	3	5.17	22	1	4.55	208	4	1.92	3	0	0.00
São João do Iracema	11	63	0	0.00	495	6	1.21	32	2	6.25	210	0	0.00	5	1	20.00
Marinópolis	12	674	8	1.19	1	0	0.00	14	2	14.29	5	0	0.00	527	4	0.76
Monte Azul Paulista	13	152	0	0.00	92	0	0.00	58	0	0.00	96	0	0.00	4	0	0.00
Paraíso	14	58	0	0.00	32	2	6.25	22	2	9.09	7	0	0.00	4	1	25.00
Guapiaçu	15	290	1	0.34	48	0	0.00	251	0	0.00	15	1	6.67	6	0	0.00
Nova Granada	16	50	0	0.00	128	0	0.00	235	1	0.43	41	0	0.00	115	2	1.74
Orindiuva	17	195	0	0.00	17	0	0.00	1494	0	0.00	9	0	0.00	540	0	0.00
Palestina	18	38	0	0.00	5	0	0.00	796	2	0.25	6	0	0.00	47	0	0.00
	19	281	4	1.42	159	1	0.63	1338	18	1.35	141	0	0.00	142	0	0.00
Américo de Campos	20	412	3	0.73	144	1	0.69	244	6	2.46	118	1	0.85	15	1	6.67
Riolândia	21	52	6	11.54	538	3	0.56	596	19	3.19	279	0	0.00	70	0	0.00
	22	73	2	2.74	163	4	2.45	292	12	4.11	101	1	0.99	22	0	0.00
Pontes Gestal	23	794	10	1.26	143	0	0.00	153	4	2.61	80	1	1.25	45	0	0.00
Cardoso	24	376	20	5.32	2	0	0.00	20	1	5.00	7	0	0.00	2	0	0.00
	Total	8724	109	1.25	2085	22	1.06	6495	95	1.46	1459	9	0.62	2661	12	0.45

NHP: non-human primate; Col.: females collected, Eng.: females engorged and %E/C: percentage of females engorged out of all females collected.

*Aedes serratus* (G): Group including *Ae. serratus* and *Ae. serratus/aenigmaticus*. Others corresponding to: *Ae. fulvus*, *Ae. terrens*, *Coquillettidia venezuelensis*, *Haemagogus janthinomys/capricornii*, *Mansonia humeralis*, *Ma. titillans*, *Psorophora discrucians* and *Wyeomyia spp.*

Of the 245 females engorged, eight did not react to any type of antibody used (3.27%). For the others, from one to five different blood sources were identified in each specimen, with predominance of three (n = 86, 35.10%) and lesser frequencies of five sources (n = 3, 1.22%). Samples with two (n = 62) or four (n = 63) different sources represented 25.31 and 25.71% of the total, respectively, and with only one blood source (n = 23), 9.39%. Among the most abundant species, the percentage for one source was of 11.92% for *Ae. scapularis*, 7.37% for *Ps. albigenu*, 5.00% for *Ae. serratus* (Group) and 11.11% for *Ps. ferox.* Only primates, NHP and bovine were identified in isolation. In the remaining samples 18 combinations of different types of blood sources were observed (Table 2).

**Table 2:** **Number of samples by combinations and numbers of types of blood source**

**Blood Source**	**Species**											
	***Ae. scapularis***		***Ae. serratus*** **(G)**		***Ps. albigenu***		***Ps. ferox***		**Others**		**Total**	
	**n**	**%**	**n**	**%**	**n**	**%**	**n**	**%**	**n**	**%**	**n**	**%**
Primates	8	7.34	0	0.00	5	5.26	0	0.00	0	0.00	13	5.31
Primates + Bird	22	20.18	1	5.00	17	17.89	0	0.00	3	25.00	43	17.55
Primates + Bird + Cow	18	16.51	6	30.00	18	18.95	1	11.11	3	25.00	46	18.78
Primates + Bird + Horse	0	0.00	0	0.00	2	2.11	0	0.00	0	0.00	2	0.82
Primates + Cow + Horse	0	0.00	0	0.00	1	1.05	0	0.00	0	0.00	1	0.41
Primates + Bird + Cow + Horse	9	8.26	5	25.00	29	30.53	0	0.00	0	0.00	43	17.55
Primates + Bird + Cow + Rat	4	3.67	1	5.00	2	2.11	0	0.00	0	0.00	7	2.86
Primates + Cow + Horse + Rat	1	0.92	0	0.00	0	0.00	0	0.00	0	0.00	1	0.41
Primates + Bird + Cow + Horse + Rat	2	1.83	0	0.00	1	1.05	0	0.00	0	0.00	3	1.22
Human + Bird + Cow + Horse	1	0.92	0	0.00	0	0.00	0	0.00	0	0.00	1	0.41
NHP	2	1.83	1	5.00	2	2.11	0	0.00	1	8.33	6	2.45
NHP + Cow	7	6.42	2	10.00	5	5.26	2	22.22	1	8.33	17	6.94
NHP + Bird + Cow	12	11.01	2	10.00	4	4.21	0	0.00	0	0.00	18	7.35
NHP + Bird + Horse	1	0.92	0	0.00	0	0.00	0	0.00	0	0.00	1	0.41
NHP + Cow + Horse	12	11.01	0	0.00	2	2.11	3	33.33	0	0.00	17	6.94
NHP + Cow + Rat	0	0.00	0	0.00	1	1.05	0	0.00	0	0.00	1	0.41
NHP + Bird + Cow + Horse	1	0.92	2	10.00	3	3.16	1	11.11	1	8.33	8	3.27
NHP + Bird + Cow + Rat	4	3.67	0	0.00	0	0.00	0	0.00	0	0.00	4	1.63
Bird + Cow	0	0.00	0	0.00	1	1.05	0	0.00	0	0.00	1	0.41
Cow	3	2.75	0	0.00	0	0.00	1	11.11	0	0.00	4	1.63
Cow + Horse	1	0.92	0	0.00	0	0.00	0	0.00	0	0.00	1	0.41
1 source	13	11.93	1	5.00	7	7.37	1	11.11	1	8.33	23	9.39
2 sources	30	27.52	3	15.00	23	24.21	2	22.22	4	33.33	62	25.31
3 sources	43	39.45	8	40.00	28	29.47	4	44.44	3	25.00	86	35.10
4 sources	19	17.43	8	40.00	34	35.79	1	11.11	1	8.33	63	25.71
5 sources	2	1.83	0	0.00	1	1.05		0.00	0	0.00	3	1.22
Not reactive	2	1.83	0	0.00	2	2.11	1	11.11	8	66.67	8	3.27
Total	109	100	20	100	95	100	9	100	12	100	245	100

NHP: non-human primate.

*Aedes serratus* (G): Group including *Ae. serratus* and *Ae. serratus/aenigmaticus*. Others correspond to: *Ae. fulvus*, *Ae. terrens*, *Coquillettidia venezuelensis*, *Haemagogus janthinomys/capricornii*, *Mansonia humeralis*, *Ma. titillans*, *Psorophora discrucians* and *Wyeomyia spp.*

Primate blood was in general predominant, being found in 94.29% of the samples (n = 231), with 64.50% for primates (n = 158), 0.41% for humans exclusively (n = 1) and 29.39% for NHP exclusively (n = 72). Bird and bovine were practically equal, being present in 176 (71.84%) and 172 (70.20%) samples, respectively. Equine blood was observed in 77 specimens (31.43%) and rat blood, the least frequent source, in 16 females (6.53%) (Table 3).

**Table 3:** **Number of samples by type of blood source, collection point and river basin**

**Blood source**	**River basin/Collection site**														
	**Tietê**										**São José dos Dourados**				
	**1**	**2**	**3**	**4**	**5**	**6**	**7**	**8**	**Sub-T**		**9**	**10**	**11**	**12**	**Sub-T**
Primate	4	7	7	0	0	1	9	0	**28**		33	2	6	5	**46**
Human	0	0	0	0	0	0	0	0	**0**		0	0	0	0	**0**
NHP	0	0	0	0	0	0	0	0	**0**		8	15	2	5	**30**
Bird	4	7	7	0	0	1	9	0	**28**		28	4	6	3	**41**
Cow	0	1	4	0	0	2	7	1	**15**		11	17	7	5	**40**
Horse	0	0	1	0	0	0	3	1	**5**		0	15	3	1	**19**
Rat	0	0	0	0	0	0	0	0	**0**		0	0	0	0	**0**
Not reactive	1	0	0	0	0	0	1	0	**2**		2	0	1	3	**6**
Number of Engorged ♀	**5**	**7**	**7**	**0**	**0**	**3**	**10**	**1**	**33**		**43**	**18**	**9**	**14**	**84**
**Blood source**	**River basin/Collection site**													**Total**	
	**Turvo-Grande**														
	**13**	**14**	**15**	**16**	**17**	**18**	**19**	**20**	**21**	**22**	**23**	**24**	**Sub-T**		
Primate	0	3	1	2	0	2	18	9	22	13	11	3	**84**	**158**	
Human	0	0	0	0	0	0	0	0	0	0	0	1	**1**	**1**	
NHP	0	0	1	1	0	0	5	3	6	6	4	16	**42**	**72**	
Bird	0	3	2	3	0	2	22	9	24	14	10	18	**107**	**176**	
Cow	0	3	2	3	0	2	21	11	24	18	13	20	**117**	**172**	
Horse	0	0	1	1	0	0	9	5	24	6	6	1	**53**	**77**	
Rat	0	0	0	0	0	0	6	1	1	3	3	2	**16**	**16**	
Not reactive	0	0	0	0	0	0	0	0	0	0	0	0	**0**	**8**	
Number of Engorged ♀	**0**	**3**	**2**	**3**	**0**	**2**	**23**	**12**	**28**	**19**	**15**	**21**	**128**	**245**	

NHP: non-human primate; ♀ = females.

**Figure 2:**
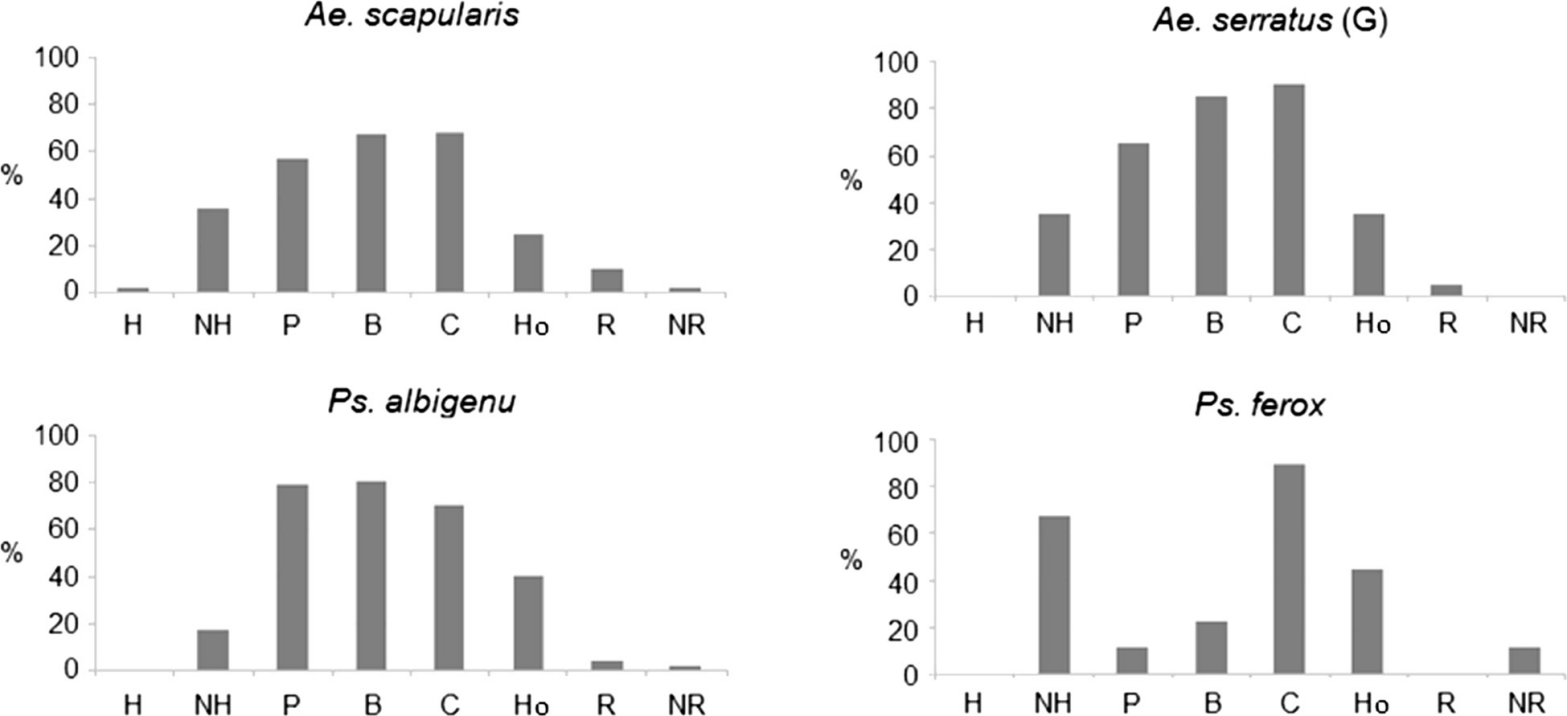
**Percentages of blood-source types for most abundant species of the total processed, by species.** H: human (exclusive), NH: non-human primate (exclusive), P: primate (human + non-human), B: bird, C: cow, Ho: horse, R: rat and NR: not reagent. *Aedes serratus* (G): group including *Ae. serratus* and *Ae. serratus/aenigmaticus*.

Among the most abundant species, *Ps. albigenu* presented a larger number of multiple sources, proportionally, than did *Ae. scapularis*, the majority being of four different origins. The difference between primates and NHP was also greater in *Ps. albigenu* than in *Ae. scapularis*, with a greater proportion of co-occurrence of human and monkey for the first specie. Bird blood occurred with greater frequency in *Ps. albigenu* and *Ae. serratus*; bovine blood was proportionally most frequent in *Ps. ferox* and *Ae. serratus. Psorophora ferox* was also notable for presenting the greatest frequency of NHP blood and the lowest frequency of human blood among the more abundant species (Figure 2). The only specimen of *Hg. janthinomys/capricornii* presented primate, bird and bovine blood, the most frequent combination among all the samples.

As to the sample distribution by hydrographic basin and collection site (Table 3), the greatest portion of them were obtained in the São José dos Dourados basin (n = 84) and at sites 19 to 24 of the Lower Turvo basin (n = 118). Sites with no samples occurred in the basins of the Tietê (4 and 5) and Middle and Upper Turvo (17 and 13, respectively).

In relation to the identification of sources, only rat blood was not found in samples from the Lower Turvo, which presented greater proportions of samples with equine (n = 51; 66.23%), bovine (n = 107; 62.21%), bird (n = 97; 52.11%) and human blood (n = 77; 48.43%); the frequency of source NHP was greater at these sites, though proportionally lower than the São José dos Dourados basin if the number of collection sites is taken into consideration (Table 3).

When the findings from each collection site are assessed, it may be noted that birds, bovines and primates alternated in the position of most frequent source at all the sites. Site 10 is noteworthy mainly for the quantity of samples with NHP and equine blood, and the low frequency of that of birds and humans. A relatively greater abundance of NHP exclusively was also found at site 24, with low human occurrence. Only at site 9 did the samples with human blood exceed the quantity of those from some other blood source. It is noteworthy that the human and bird sources were not detected in samples of the site 8. Bovine blood was absent only at site 1, whereas equine blood was identified at 14 of the 20 sites at which engorged females were obtained; rat blood was found in samples from six different localities and non-reactive samples in five. NHP exclusively was registered at 12 of the 20 localities with samples (Table 3).

## Discussion

The presence of engorged females is associated with microhabitats of resting places and digestion of blood, which do not necessarily coincide [28]. The feeding habit can be modified by geographical variations among distinct subpopulations or by behavioral changes in the same subpopulation within one single reproductive season [29]. Some Culicidae species bite one or more hosts repeatedly during each gonotrophic cycle, while others are less active [25]. There are cases in which this may also be observed within the same species under the influence of meteorological variations, by infection with some parasite or even by the relative size of the specimens [30-32]. In addition, the influence of the chosen types of collection methods must always be appreciated in the discussion of results [33,34].

In this present study, the only collection technique used was applied at ground level, in forest fragments, the majority of which presented smaller areas compared to studies on fragmented landscapes [23]. The canopy heights of about 70% of these fragments attained less than fifteen meters (Additional file 1), being considered relatively low as compared to the heights established for the installation of arboreal platforms for collections in Amazonia, where the tops of the trees commonly reach 30 to 40 meters [35]. Nevertheless, some studies conducted in forest environments of the Savannah and Atlantic Forest biomes have shown more flexible behavior on the part of these mosquito species in their selection of the heights of the tree strata at which they undertake their egg-laying and their feeding on hosts [16,36].

In view of this aspect, it was decided to apply the sampling effort to a horizontal rather than a vertical plane. According to the environmental characteristics of the study area, we considered that the collection technique whereby the collector moves around (allowing the generation of a series of intrusion effects) results in the coverage of a more varied gradient of microhabitats than the stationary technique on canopy platforms. Furthermore, distances of up to 1,100 meters, from the edge to the interior of the forests, were covered.

Despite this, no male specimens of *Hg. janthinomys* or *Hg. capricornii* were obtained, which made it impossible to determine which of the two species was more frequent in the area. In previous research undertaken in the same region, the collection of immature forms in the hollow spaces in trees in the municipality of Santa Albertina (SP, Brazil) permitted the identification of males of *Hg. janthinomys*, while *Hg. capricornii* was registered toward the southeast, in the municipality of Araraquara (SP, Brazil) [37,38].

It is worth observing that collection sites with the greatest relative abundances of *Hg. janthinomys/capricornii* showed a proximity of about 25 kilometers (8 to 45 km) from the locations of the epizootic outbreaks of *Allouatta caraya* confirmed in 2008 [14,21]. At these sites, the forest fragments presented the greatest canopy heights (of about 25 m) and the largest areas of all the collection sites. In that setting the only engorged female of *Hg. janthinomys/capricornii* found was one of five specimens caught at site 20 and presented bird, primate and bovine blood, showing eclecticism as well as affinity for human blood, a fact also observed in relation to *Hg. janthinomys*, but not *Hg. capricornii* [15,16]. The forest fragment at that same location, one of the smallest, having an area of 12.1 hectares, was where *Ae. scapularis*, *Ae. serratus/aenigmaticus* and *Ps. albigenu* were found with NHP blood (Additional file 1).

A synthesis of these results suggests that even though *Hg. janthinomys/capricornii* is well-adapted to better preserved wild areas, it frequents the degraded, fragmented environment of the region, maintaining its population on the basis of an original re-colonization of better preserved forests. This, together with the functional connectivity of these landscapes, reproduces dispersion systems already reported for other animal groups [39].

Furthermore, the simultaneous finding of a female of *Hg. janthinomys/capricornii* engorged with human and monkey bloods and of three other mosquito species with monkey blood in one of the smallest forest fragments indicates the possibility of the transmission of sylvatic yellow-fever in highly modified environments, a behavior also presented by ticks and Brazilian spotted fever in the Atlantic Forest and also discussed in relation to the epizootic outbreaks that occurred in São Paulo state in 2008/2009 [20,40]. That is to say, reflecting a process opposite to that of a dilution effect, by which the probability of less specific contacts between vectors and hosts is increased by the fragmentation of their habitat [41]. The high percentage of multiple blood sources found in engorged females of the present study corroborates that model.

The presence of only one blood source type in merely 9.39% of the specimens is much lower in comparison with the results of other research projects on wild species. Alencar *et al.* [15] found 60.6% of the females of *Hg. janthinomys* with only one blood source in 11 localities of forested environments in five Brazilian states. In four well-preserved forested localities in central and southeastern Brazil, Alencar *et al.* [16] found 68.3% of the females of *Hg. leucocelaenus* and 66.7% of those of *Hg. capricornii* in the same condition. Moreover, dos Santos Silva *et al.* [42] – in six localities situated in units of environmental conservation, distributed in the Atlantic Forest, Pantanal and Cerrado biomes – found 74.7% of the females of 15 different Culicidae species with only one blood source.

Day [43] defined three strategies in the feeding behavior of the culicids: directed search, co-habitation and intense eclecticism. Despite *Ae. scapularis* and *Ps. albigenu* appearing to come under the third definition, it is supposed to not be an intrinsic characteristic of that species, because it has also been observed among those less frequent ones. On analyzing patterns of vector/host co-occurrence for a series of localities, Chaves *et al.* [19] concluded that the contact between a particular mosquito species and a host of a particular bird or mammal species depended more on the presence/absence of that host than on any intrinsic choice on the part of the vector.

The predominance of bird blood was observed by dos Santos Silva *et al.* [42] in all the 15 species of Culicidae obtained, some of them, such as *Ae. fulvus*, *Ae. scapularis, Ae. serratus, Ps. albigenu*, *Ps. ferox* and *Ma. titillans* having been investigated in this present study. For *Hg. janthinomys,* in nine out of 11 localities distributed throughout Brazil, the predominance of bird blood has been registered, with rodent and human blood being of secondary importance [15]. In the state of Rio de Janeiro, *Hg. capricornii* and *Hg. leucocelaenus* also present a preference for birds, though in Goiás, rodents, humans and marsupials represent important sources for *Hg. leucocelaenus* [16].

In the Ribeira Valley, in the southern portion of the state of São Paulo, research in five localities that formed an environmental gradient of agricultural and cattle-raising activity, including residual Atlantic Forest, showed that only in *Ae. serratus* was bird, bovine and human blood found in the same proportions, with some tendency toward an association with birds in more highly preserved environments. For *Ae. scapularis,* bovine blood was predominant, followed by equine and human (primate) and for *Ps. albigenu* and *Ps. ferox*, human (primate) blood [44,45].

In the light of these findings, it may be inferred that birds serve as constant and frequent hosts for many Culicidae species, whereas the use of other blood sources, especially when predominant, depends more on specific situations. The idea that the finding of a particular blood source in female mosquitoes is the result of a balance between adaptive feeding affinity, the opportunity offered by the host and furthermore, on local environmental conditions, as has been observed in North American mosquitoes [33,34].

When the four most abundant species are considered, it may be noted that only in *Ps. ferox* was bird blood relatively rare, whilst bovine blood was predominant. For *Ae. scapularis, Ae. serratus* and *Ps. albigenu*, bovines also represent an important source when the different collection sites are considered. As to the availability of these hosts, it was observed that in all localities where pastures and installations associated with cattle production and management were present, engorged females were obtained with this source. An important aspect to consider is that the invasion of the natural areas by bovine cattle is commonly observed in the state of São Paulo [46]. From an epidemiological approach, the role of large mammals has been discussed not only as to their importance as a reservoir but also in relation to zooprophylaxis and zoopotentiation [47,48].

As to the primates, the group of greatest interest for the understanding of the wild cycle of yellow fever virus, coincidentally fewer NHP blood samples were detected at the sites closest to the epizootic area (1 to 6 localities) [14,21]. Conversely, at location 10, which corresponded to the single unit of environmental conservation among all sites of collection, the proportion of monkey blood samples was the greatest. Furthermore, no monkey deaths were registered in the municipality (Figure 1), which may constitute evidence of a dilution effect [42].

The fact that the human blood was found in samples from all the collection sites, except one, demonstrates the close proximity of humans to the wild ecotone. In five of these localities the residential estates were adjacent to the forest fragments while at seven sites the farmhouses were located less than 800 meters from the forested areas. Ten of the localities lay beside great reservoirs while the others were situated on the banks of large rivers (Additional file 1). All these areas present attractions commonly used by the local population for recreational, sporting and fishing activities or for extracting clay and sand. The latter situation illustrates the occurrence of the autochthonous human case in São Carlos (SP, Brazil) in 2008 [21].

These aspects reinforce the need to maintain continuous assessments of vaccination coverage [22]. On the other hand, considering the most abundant species collected during this study, it is also recommended that further research should be undertaken to ascertain other aspects of the vector capacity and competence of secondary vectors of the yellow fever virus.

## Conclusions

In this study we observed a considerable proportion of non-human primate blood sources in mosquito species that show minor importance in yellow-fever virus transmission, which suggests that more attention should be dedicated to these species, especially in further studies on their vector competence and capacity.

Our results have also confirmed the proximity to or presence of human beings in the forest fragments of the collection sites at which samples were obtained. This finding indicates that the human population of the region frequents the wild ecotone, which indicates the need for periodic assessments of vaccination coverage against yellow fever in this region of São Paulo state.

Finally, the finding of such highly eclectic feeding habits of mosquitoes in an area of ancient fragmentation in the Mata Atlântica and Brazilian Savannah may serve as a warning about the establishment of new co-evolution processes involving the vectors, hosts and viruses involved in the transmission of yellow fever and other arboviruses, which may consequently challenge us to revise our risk perceptions of these diseases.

## Additional files

Additional file 1:
**Basic information on collection sites.**
Additional file 2:
**Number of collected females and males of Aedini, Mansoniini and Sabethini tribes in the northwestern São Paulo state, Brazil, between 2006 and 2008.**
Additional file 3:
**Number of collected and engorged females of the less abundant species.**
